# Characterization of Shrink Film Properties for Rapid Microfluidics Lab-on-Chip Fabrication

**DOI:** 10.3390/mi15030308

**Published:** 2024-02-23

**Authors:** Tian Fook Kong, Alger Wai Jiat Ang, Marcos

**Affiliations:** School of Mechanical and Aerospace Engineering, Nanyang Technological University, 50 Nanyang Avenue, Singapore 639798, Singapore; tianfook@ntu.edu.sg (T.F.K.); alge0002@e.ntu.edu.sg (A.W.J.A.)

**Keywords:** shrink film, microfluidics, lab-on-a-chip, wettability, hydrophobic, hydrophilic, baking temperature

## Abstract

Shrink film is a thin sheet of polystyrene plastic that shrinks to 25–40% of its original size when heated. This study investigated the shrinkage factor of the film at different temperatures and baking times to determine the optimal fabrication recipe for shrink film microfluidic device production. Additionally, this study characterized the properties of shrink film, including minimum possible feature size and cross-section geometries, using manual engraving and the CAMEO 4 automated cutting machine. The optimal shrinkage factor ranged from 1.7 to 2.9 at 150 °C and a baking time of 4 min, producing the ideal size for microfluidic device fabrication. The X- and Y-axes shrank ~2.5 times, while Z-axis thickened by a factor of ~5.8 times. This study achieved a minimum feature size of 200 microns, limited by the collapsing of channel sidewalls when shrunk, leading to blockages in the microchannel. These findings demonstrate the feasibility and versatility of using shrink film as a cost-effective and efficient material for the rapid fabrication of microfluidic devices. The potential applications of this material in various fields such as the medical and biomedical industries, bacteria and algae culture and enumeration are noteworthy.

## 1. Introduction

Microfluidics involves the examination and control of minute quantities of fluids, typically in the microliter or nanoliter range. This field has gained significant prominence in research due to its diverse applications in areas like biotechnology, pharmaceuticals, diagnostics, lab-on-chip (LOC), separation and isolation of circulating tumor cells, sperm sorting, and bacteria or algae detection [1,2,3,4,5,6,7,8,9,10,11,12,13,14,15]. The integration of microscale structures on a single chip has revolutionized research across various domains by enabling the swift manipulation, analysis, and distribution of small samples. Thus, there has been a surge in interest in the rapid fabrication of microfluidic LOC devices, as it combines automation and miniaturization to produce highly precise gadgets quickly. One approach for this rapid fabrication is using shrink films.

Shrink films offer exceptional utility (Table 1) in LOC development because they can be rapidly produced and designed with features as thin as hundreds of microns [16]. Customizable features on shrink films allow for the swift design, production, and assembly of complex microfluidic LOC systems. The potential applications of shrink-film-based rapid microfluidic LOC fabrication have garnered attention in various domains [17]. However, there is a noticeable gap in research when it comes to assessing the reproducibility and scalability of shrink-film-based fabrication processes despite their apparent advantages. Although prior studies have demonstrated the feasibility of using shrink film for quick prototyping and cost-effective microfluidic device production [16,17], there is a need for more rigorous and quantitative analysis of the resulting devices. This includes a closer examination of the impact of process variables like temperature and time. Addressing this research gap can offer valuable insights into the practical applications of shrink-film-based microfluidic LOC devices and provide avenues for optimization and innovation.

In this paper, we present an extensive investigation into shrink film microfluidics and its potential impact on LOC fabrication and the broader field of microfluidics. Specifically, we explore the characteristics of shrink film for rapid microfluidic LOC fabrication and evaluate the consistency and reliability of shrink-film-based microfluidic devices. Our study accomplishes the following objectives: Firstly, a parametric study was conducted to examine the shrinkage factor before and after exposure to various temperatures and baking times to identify an optimal fabrication process. Secondly, properties such as the minimum feasible feature size and cross-sectional geometries using different fabrication methods, including manual engraving and an automated paper-cutting machine, were characterized. And thirdly, the usability of shrink film in creating microfluidic devices was assessed through two methods: (a) directly utilizing the shrink film itself as the microchannel and thermally bonding it with another piece of blank shrink film; and (b) using shrink polystyrene film as a mold for PDMS casting and fabrication. 

## 2. Experimental Section

### 2.1. Materials

We investigated the characteristics and fabrication of a shrink film microfluidic device using a polystyrene (PS)-based shrink film (Shrinky Dinks, Alex Brands, Fairfield, NJ, USA). The shrink film was cut into rectangular pieces with dimensions of 20 mm by 30 mm using an automated paper-cutting machine (Cameo 4, Silhouette America, Lindon, UT, USA). The original thickness of the shrink film measured from 48 pieces of shrink film was approximately 272 ± 4 µm.

### 2.2. Heating Method

To identify the most appropriate heating equipment for heating the shrink film, three types of equipment were evaluated: a hot plate (C-MAG HS 4, IKA, Wilmington, NC, USA), a handheld heat gun (Model 6519, 2500 W, Lo’Master, Shenzhen, China), and a convection oven (TO 977SS, 9 L, 700 W, Toyomi, Singapore). All three types of equipment were set to operate at a temperature of up to 250 °C to evaluate their suitability.

### 2.3. Baseplate Material

We assessed the suitability of both glass slides and steel plates as potential substrate base materials for the shrink film to be placed on during the heat shrink process. Ethanol was used to clean the surfaces of both materials and eliminate any contaminants. A piece of shrink film was placed on the base plate surface and heated in an oven at 90–250 °C for up to 10 min. After heating, the shrink film was observed for any signs of surface adhesion, distortion or curling.

### 2.4. Fabrication of Shrink Film Microfluidic Chip

**Automated paper-cutting machine.** The microfluidic channel design was created using computer-aided design (CAD) software (SolidWorks 2023, Waltham, MA, USA). The CAD design was then exported in a vector format, DXF, to an automated paper-cutting machine. CAMEO 4 was used to cut the microchannels, reservoirs, and other features into the shrink film. This method allows for the regulation of depth and width to suit specific requirements, making it a favorable and cost-effective approach to creating microfluidic channels with various dimensions and shapes. The shrink film with microchannel embedded was then heated to a blank piece of shrink the film base to create a sealed microfluidic chip.

**Dremel/engraving pen.** Creating channels on shrink film using a Dremel pen involves manually inscribing the microfluidic channel design onto the shrink film using the Dremel pen. The engraving pen has a small, sharp tip that can be used to create lines and shapes. The user would need to have a steady hand and good control to ensure the lines are straight and accurate. This method may be more time consuming and prone to errors compared to using design software and cutting with a machine, but it can be a useful option for small-scale or one-off fabrication. 

**Procedures.** Once the channel is created, the chip is assembled by fusing two pieces of shrink film to seal the channel surface. This method offers an efficient and cost-effective solution to the production of microfluidic devices. The fabrication process of a microfluidic device with shrink film involves the following steps. Firstly, the microfluidic channel design is designed using computer-aided design (CAD) software such as SolidWorks, and then exported as a DXF file. Subsequently, the DXF file is imported into Silhouette Studio, a software compatible with the CAMEO 4 machine, for cutting preparation. Subsequently, the shrink film is placed and secured onto the cutting mat. The CAMEO 4 machine is configured to the appropriate settings for inscribing, encompassing parameters like cutting speed and passes. Once set up, the cutting mat with the shrink film is loaded into the machine, initiating the inscribing process. Post cutting, the shrink film undergoes thorough cleaning with ethanol to eliminate any residual debris or dust. Next, the oven is preheated to 150 °C, and the shrink film is positioned on a steel plate, heated for 4 min, and allowed to cool. Sequentially, another layer of shrink film is positioned atop the inscribed film. Both layers are then heated together in the oven, with the aid of a steel block to ensure uniformity and flatness, thereby effectively fusing them. This process results in a microfluidic device made entirely of shrink film, with a sealed channel for fluid flow.

### 2.5. Alternative Method of Using Shrink Film as Mold for PDMS Microfluidic Chip Fabrication

This process involves the creation of a patterned shrink film using CAD and CAMEO 4 to cut out the microchannels’ pattern, which are subsequently used as a mold to cast a polydimethylsiloxane (PDMS) chip. The use of this process eliminates the need for the traditional photolithography method, ultimately leading to reduced costs and fabrication time. This approach offers a promising alternative to the existing fabrication process, providing a simpler and more cost-effective method to produce microfluidic devices. The fabrication process of a microfluidic device with PDMS utilizing shrink film as a mold involves the following steps: Firstly, the microfluidic channels are designed using SolidWorks and exported as a DXF file. Subsequently, the DXF file is imported into Silhouette Studio, where the channel design is cut onto a shrink film using the CAMEO 4 machine, followed by cleaning the film with ethanol. The next step involves preheating the oven to 150 °C and placing the shrink film mold on a steel plate, heating it for 4 min before allowing it to cool. Glue is then applied to the bottom surface of the mold, adhering it to a Petri dish to prevent movement. Next, the required amount of PDMS is determined and mixed at a 10:1 ratio of PDMS base to curing agent. The mixture is then poured into the Petri dish and spread evenly to achieve the desired thickness. Subsequently, the Petri dish is placed in a vacuum chamber to degas the PDMS mixture and remove air bubbles before allowing it to cure. Once cured, the PDMS is gently peeled off the mold or substrate. Bonding the PDMS chip to a glass slide is accomplished using a plasma machine, followed by creating inlets and outlets in the PDMS using a biopsy punch. Finally, the microfluidic device is tested by introducing fluids into the inlet and observing the flow through the channel to validate its functionality. The result of these steps is a functional microfluidic device made of PDMS.

### 2.6. Creating of Chip Holder for Mounting of Microfluidic Device

The holder’s design can be tailored to incorporate features like fluid inlet ports and waste disposal outlets. Selecting an appropriate holder will hinge on the experimental requirements, including the intricacy of the microfluidic device’s design and the experimental conditions. For example, we designed a shrink film engraved with microfluidic channel design measuring 80 mm by 40 mm. After considering the shrinkage ratio, the final shrunk size of the film in the X- and Y-axes can be determined. It is important to design the holder in such a way that it can enclose a microfluidic device with dimensions of 35 mm by 17.5 mm, which were obtained through the calculation of shrinkage factors. The dimensions of the holder will also need to be adjusted to accommodate any additional features such as ports and outlets required for the specific experiment. Overall, ensuring that the dimensions of the holder are accurately designed is crucial to providing a stable platform for the microfluidic device during experimental use.

## 3. Results and Discussion

### 3.1. Heating Method

**Figure 1:**
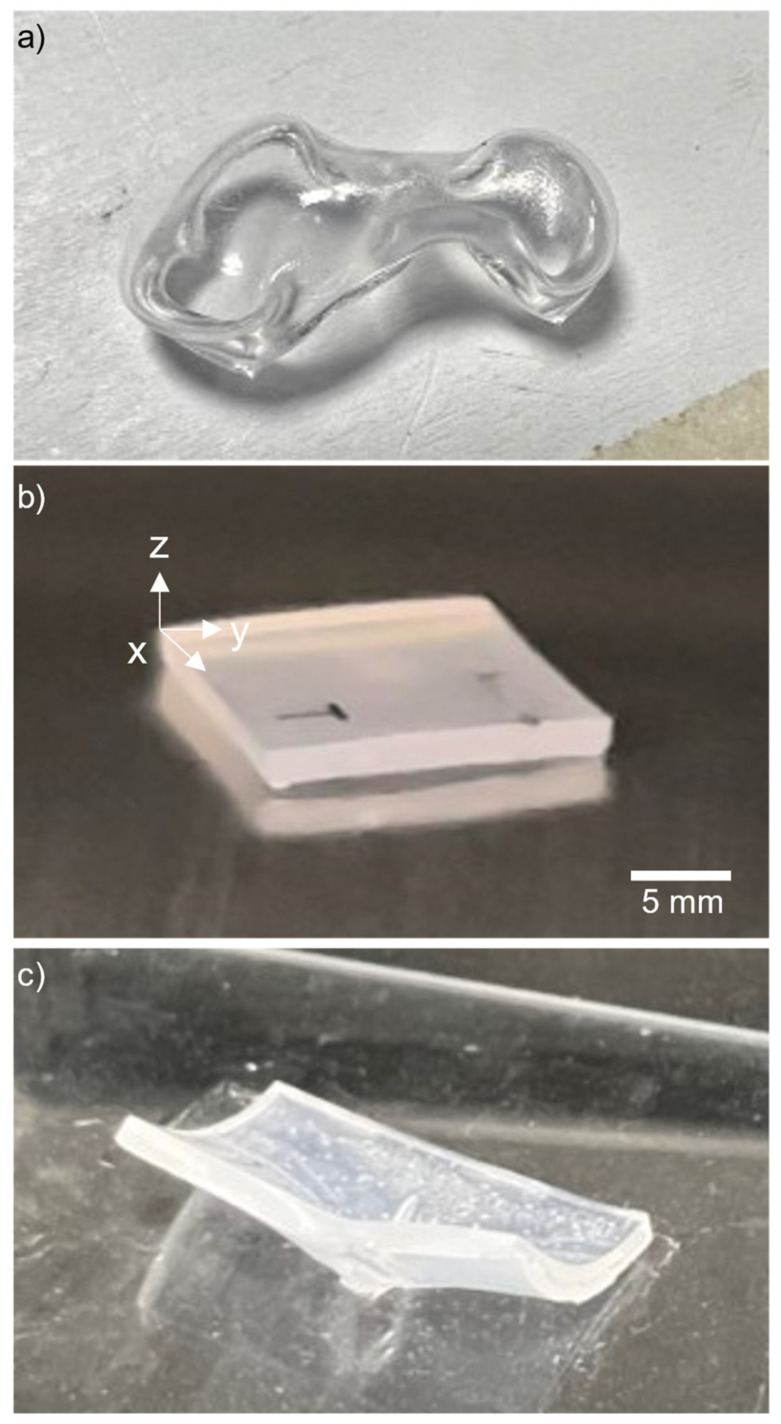
The shrink film exhibited different behaviors under various heating conditions: (**a**) when subjected to heat from both a hot plate and a heat gun, the film curled due to localized heating. In contrast, (**b**) when the film was heated in an oven positioned on a stainless-steel plate, it flattened out uniformly because the oven provided more even heating across the entire film; additionally, a steel block can be placed on the top surface of the shrink film to further flatten the geometry; and (**c**) conversely, when the film was placed on a glass slide as a substrate in oven, it curled at the edges, while a portion of the film adhered to the glass in the middle, resulting in an unsuccessful shrinking procedure.

To identify the most appropriate heating equipment for heating the shrink film, three types of equipment were evaluated: a hot plate, a handheld heat gun, and a convection oven. All three types of equipment were set to operate at a temperature of 170 °C to evaluate their suitability. During the experiment, it was observed that the shrink film curled up (Figure 1a) when heated using both the hot plate and heat gun due to the localized heating provided by these devices. In contrast, when the shrink film was heated using the oven, it flattened out (Figure 1b) after heating as the oven provided even heating throughout the shrink film. Hence, we consider that the oven proved to be the optimal equipment for manufacturing shrink film microfluidic devices owing to its reliable and even heating. In contrast to the hot plate and heat gun, which only applied heat to a surface, a convection oven featured heating elements positioned both at the top and bottom, enabling a more effective distribution of heat.

### 3.2. Base Plate Material

The selection of a suitable base material is crucial in ensuring that the shrink film can shrink satisfactorily and flatten out evenly during the heating process. As previously studied, thermal adhesion can cause the shrink film to stick to the base material surface. A steel plate and a glass plate (Figure 1c) were chosen as the two base substrate materials to be tested. The main criterion for selection was that the shrink film should not stick to the chosen material during the heating process. The glass plate was found to be unsuitable as the middle portion of the shrink film adhered to the glass surface and resulted in significant curling at the edges (Figure 1c). On the other hand, the steel plate was found to be suitable as the shrink film remained flat and did not stick to the surface during the heating process (Figure 1b). Based on these results, the steel plate was chosen as the base material for subsequent experiments.

### 3.3. Shrinking Temperature

A parametric study was conducted to determine the optimal shrinking temperature for the polystyrene film. To establish the ideal temperature, we defined the shrinkage factor, *η*, as follows:

$$\eta =\frac{Dimension\;before\;baking}{Dimension\;after\;baking}$$

**Figure 2:**
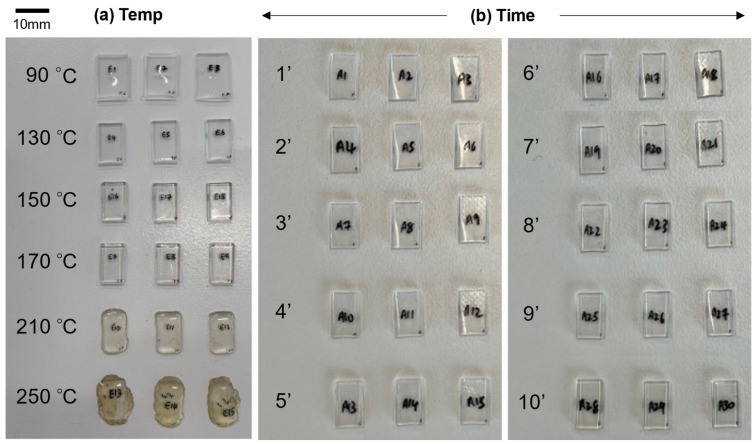
Shrink film specimens with dimensions of 20 mm by 30 mm after heating (**a**) at different temperatures (90 °C, 130 °C, 150 °C, 170 °C, 210 °C, and 250 °C) for 10 min, and (**b**) baking at different durations of time ranging from 1 to 10 min at 150 °C, and cooled to room temperature before visual inspection. Each temperature and time point are repeated three times (*N* = 3 triplicates).

The shrinkage factor was utilized to ascertain the optimal temperature, which served as a gauge for measuring the degree of shrinkage before and after baking in a convection oven. For this experiment, shrink film specimens with dimensions of 20 mm by 30 mm were prepared. These samples were heated for 10 min at temperatures of 90 °C, 130 °C, 150 °C, 170 °C, 210 °C, and 250 °C (Figure 2a). Each temperature point was repeated three times (*N* = 3 triplicates). 

**Figure 3:**
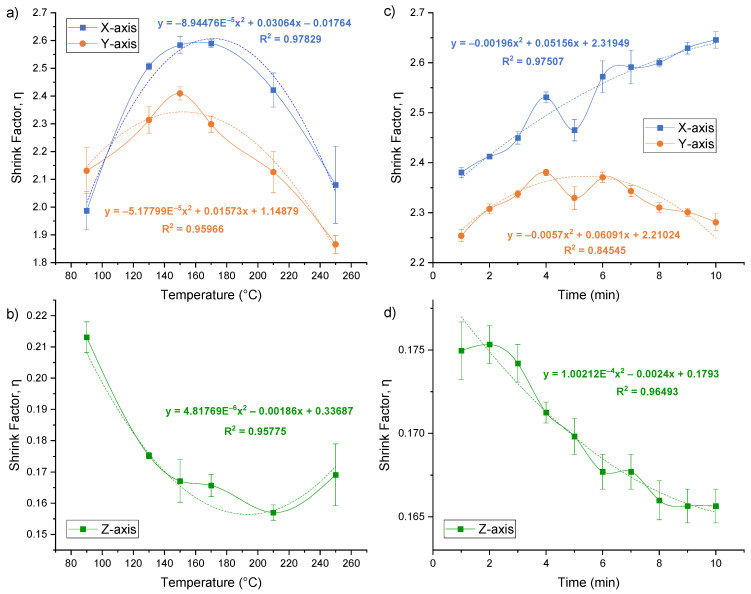
(**a**,**b**) Shrinkage factor of X-, Y-, and Z-axes plotted against temperature for shrink film specimens heated at different temperatures ranging from 90 °C to 250 °C for 10 min. The shrinkage factor increased with increasing temperature up to 150 °C and started to decrease after that point. (**c**,**d**) Shrinkage factor plotted against heating time for X-, Y-, and Z-axes of the shrink film specimens at 150 °C. For X-axis, the shrink factor increased with time, while for Y-axis, the shrinkage factor increased gradually from 1 min and peaked at 4 min of heating time, after which it reached a plateau and decreased slightly till 10 min. The dotted line represents polynomial fit, while the solid line is the scatter plot smooth curve line.

After each heating, the specimens were removed from the oven and allowed to cool to room temperature. Visual inspection and dimension measurements were carried out and revealed that the shrink film did not shrink enough at the lowest temperature of 90 °C, leading to incomplete shrinking. On the other hand, the shrink film melted and showed significant deformation and cracking at higher temperatures such as 210 °C and 250 °C. The measurements of each specimen were taken along the X-, Y-, and Z-axes, and the shrinkage factor was calculated and plotted against the temperature. As shown in Figure 3a,b, the shrinkage factor increased with increasing the temperature up to 150 °C, after which it started to decrease. This suggests that 150 °C is the point at which the shrink film begins to over-shrink. Therefore, a temperature of 150 °C was deemed more suitable for shrinking the film, as it resulted in a high shrinkage factor without causing deformation. Noticeably, while the X- and Y-axes shrinkage factor has a range of 1.85 < *η* < 2.65, there is a slight difference in the *η* for the X- and Y-axes as the shrink films are made of biaxially oriented polystyrene thermoplastic sheets [16].

### 3.4. Shrinking Time

To investigate the effect of heating time on the shrinkage factor of the film, a series of experiments were carried out using the same shrink film specimen’s specification in the previous section. The specimens were placed in an oven preheated to a temperature of 150 °C and were heated for varying times of 1, 2, 3, 4, 5, 6, 7, 8, 9, and 10 min (Figure 2b). Each time point was repeated three times (*N* = 3 triplicates). The temperature and timing were carefully controlled throughout the experiment. After each heating time, the specimens were removed from the oven and allowed to cool to room temperature. The shrinkage factor was calculated using the same method as described in the previous section.

The data obtained from the measurements were plotted as shown in Figure 3c,d. The shrinkage factor was plotted against the heating time for each axis. The data were also subjected to statistical analysis to determine if there were significant differences in the shrinkage factor at different heating times. Based on the results obtained, it can be concluded that the heating time has a significant effect on the shrinkage factor of the shrink film. For the X-axis, the shrink factor increased with time, while for the Y-axis, the shrinkage factor increased gradually from 1 min and peaked at 4 min of heating time, after which it reached a plateau and decreases slightly till 10 min. Therefore, based on these results, it is recommended to use a heating time of 4 min for the shrink film to achieve the desired level of shrinkage without causing any deformation.

### 3.5. Shrink Film Wettability

In microfluidics, the surface energy of the chip material is an important consideration in designing and fabricating the device. The choice of material and surface treatment can affect the flow of fluids and the interactions between the fluid and the material surface, ultimately impacting the performance of the microfluidic device [19]. Fluids with a contact angle, θ, measuring less than 90 degrees tend to spread across a surface and are categorized as hydrophilic, whereas those with a contact angle exceeding 90 degrees, causing them to form beads, are categorized as hydrophobic [20,21]. Surfaces with a contact angle between 150 and 180 degrees are called superhydrophobic [22]. The knowledge of a fluid’s contact angle is essential for the design and operation of a microfluidic device [22]. Hydrophobic surfaces are often used in microfluidic applications to prevent unwanted adhesion and facilitate droplet formation, while hydrophilic surfaces are desirable for promoting fluid transport through channels [23]. 

**Figure 4:**
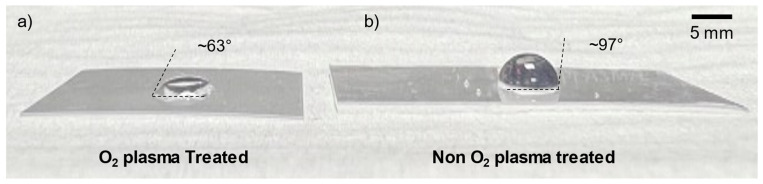
The effect of O_2_ plasma treatment on shrink film wettability pre-shrinking. (**a**) Shrink film treated with O_2_ plasma showing improved wettability with water droplets spreading out on the surface. (**b**) Untreated shrink film exhibiting hydrophobic behavior.

In order to determine the wettability of the shrink film’s surface, a droplet of water was placed onto the shrink film and the surface was visually examined to determine whether it was hydrophobic or hydrophilic. The contact angle between the water droplet surface and the shrink film surface can be estimated by image analysis with MS PowerPoint. The result shown in Figure 4b verified that the inherent surface property of the shrink film surface was indeed hydrophobic, with a contact angle of approximately 97°. Shrink film’s hydrophobic nature can be a limiting factor for certain microfluidic applications. However, there are methods to overcome this limitation. 

The wettability of a surface is one of the surface qualities of materials that can be modified using the plasma treatment procedure. The process of plasma treatment involves subjecting the material to low-pressure plasma, which can alter the surface’s chemical and physical characteristics [24]. Usually, the surface is exposed to an ionized gas, which can interact with the surface and change its properties. The construction of microfluidic devices frequently uses plasma treatment to make surfaces more hydrophilic [25]. This can be accomplished by introducing oxygen-containing functional groups onto the surface, which can increase the surface energy and thus promote wetting by aqueous solutions [26]. The extent of the surface modification can be controlled by adjusting the treatment time, gas pressure, and other parameters. Plasma treatment can be a useful tool in the development of microfluidic devices with tailored surface properties. As shown in Figure 4a, treating the shrink film with plasma (surface-treated by plasma activation; maximum RF power of 45 W for 2 min; Harrick plasma cleaner PDC-002, Harrick Plasma, Inc., New York, NY, USA) showed improved wettability, with water droplets spreading out on the surface with a contact angle of 63°, transforming the shrink film’s regime from hydrophobic to hydrophilic. 

### 3.6. Shrink Film Microfluidic Chip

**Figure 5:**
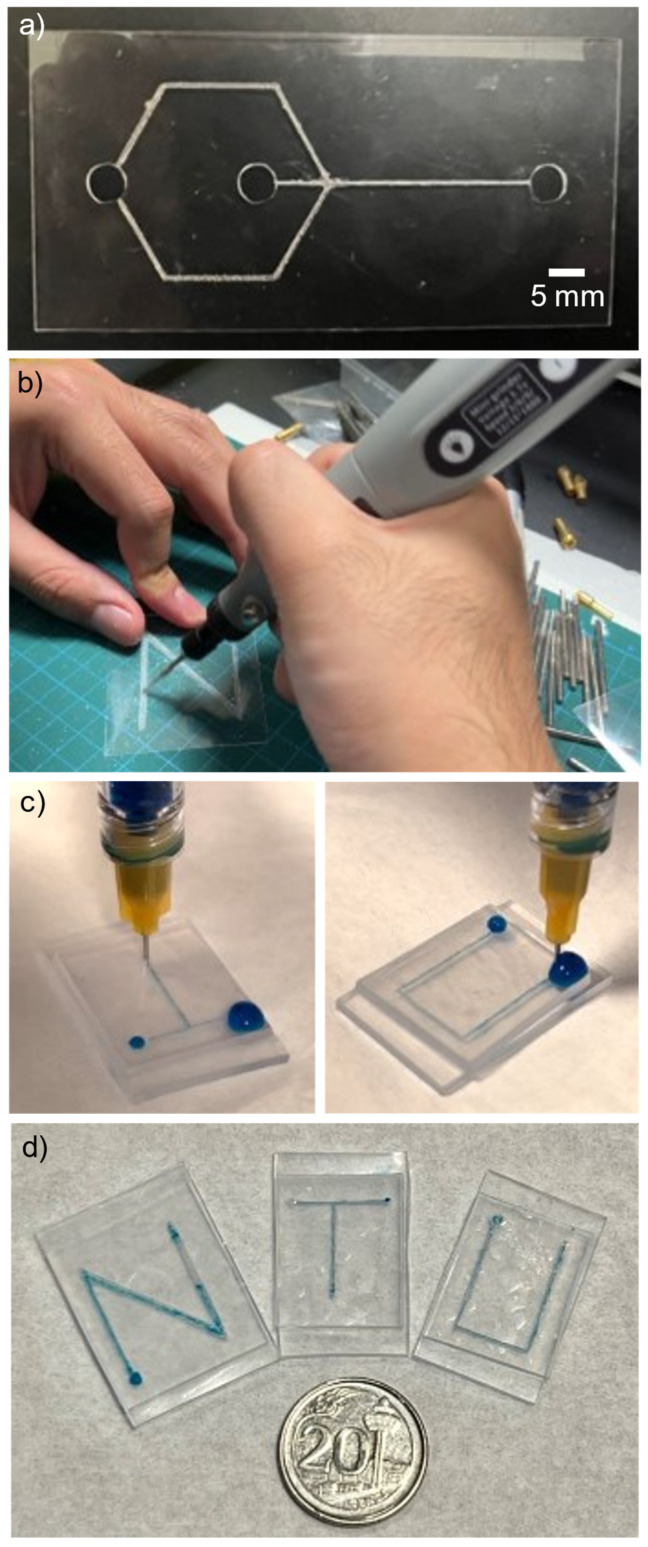
(**a**) Microfluidic channel design created by inscribing method using the CAMEO 4 paper cutter. (**b**) The microchannel pattern is manually inscribed onto a shrink film using a Dremel pen. (**c**) After shrinking and bonding of the fabricated shrink film microfluidic device, fluids were introduced into the chip’s inlets to observe the flow through the channel. (**d**) Fabricated channel using shrink film: (left) N-shaped channel, (middle) T-shaped channel, and (right) U-shaped channel.

As described in the sub-section “Fabrication of shrink film microfluidic chip” in the Experimental Section, we explored the feasibility of using the shrink film itself as the microfluidic device. Figure 5a shows a shrink film inscribed with two-inlets-one-outlet Y-shaped microfluidic channel design using a CAMEO 4 paper cutter, while Figure 5b shows an N-shaped microchannel pattern manually inscribed onto a shrink film using a Dremel pen. 

**Figure 6:**
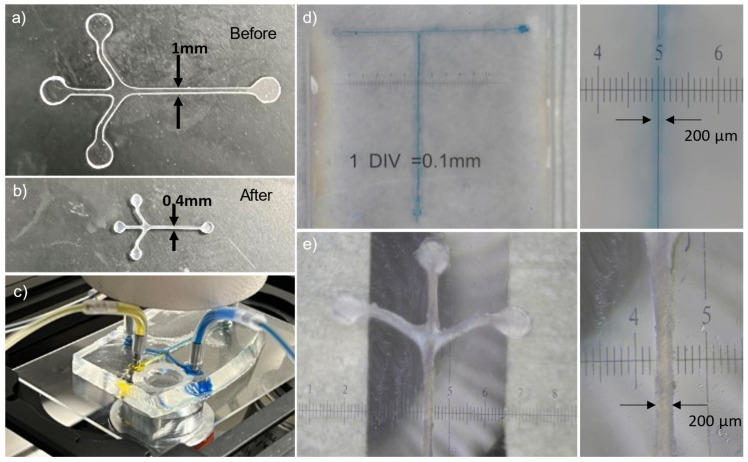
(**a**) The pre-shrunk film cut-out as a Y-shaped microchannel with channel width of 1 mm. (**b**) The shrunk microfluidic channel structure after baking the shrink film as negative impression for casting of materials such as PDMS. (**c**) Fabricated PDMS microfluidic channels (using shrink film negative impression as a mold) bonded onto a glass slide. (**d**) Minimum feature size of 200 µm for using shrink film itself as the microfluidic chip. (**e**) Minimum feature size of 200 µm for using the shrink film as negative impression for PDMS.

The results of the parametric studies in the previous sections indicated that both the baking time and temperature are critical factors that need to be optimized in the fabrication process of a shrink film microfluidic device. The optimal shrinkage factor fell within the range of 1.7 to 2.9 at 150 °C with a baking time of 4 min, yielding the ideal dimensions for microfluidic device production (Figure 3). The Z-axis thickened by a factor of approximately 5.8 times, while the X- and Y-axes underwent contraction. The unique advantage of using shrink film for a microfluidic chip is the capability to use a low-cost and conventional machining or cutting method to inscribe the microchannel onto the film with larger dimensions and lower resolution, and subsequently shrink it down by a factor of ~2.5 times. This study achieved a minimum feature size of ~200 µm (Figure 6d,e). While the paper cutter can handle geometries smaller than 0.5 mm, there is a tendency for the channel sidewalls to adhere when shrunk, leading to blockages in the microchannel.

The final step involved bonding a piece of blank shrink film as the cover layer onto the shrunken film that was engraved with microchannels through thermal bonding. A steel plate of dimension 100 mm × 100 mm × 3 mm, resulting in an approximately 2.4 N force on a post-shrunk 8 mm × 12 mm shrink film stack (with a pressure of approximately 25 kPa), is placed on top of the stacked shrink films to improve the flatness and bonding of the shrink films. The resulting structure formed a microchannel sandwiched between two layers of shrink film. By using the shrink film as the microfluidic device, the need for a separate mold, casting or additional bonding steps was eliminated, thus simplifying the fabrication process, and reducing the associated costs. Nonetheless, a drawback of shrink film microfluidic devices is that the surface roughness can impact fluid flow resistance and pressure loss along the channel length due to localized pressure buildup and the formation of eddy currents [27,28]. The effect of surface roughness could, up to some extent, be mitigated with an elevated temperature of fluid for the increased efficient flow of fluid [27].

After the shrinking and bonding of the fabricated shrink film microfluidic device, fluids (deionized water with blue dye) were introduced into the chip’s inlets to observe the flow through the channel (Figure 5c). Figure 5d exemplifies the fabricated shrink film microfluidic channels using shrink films: (left) N-shaped channel, (middle) T-shaped channel, and (right) U-shaped channel.

### 3.7. Chip Holder

**Figure 7:**
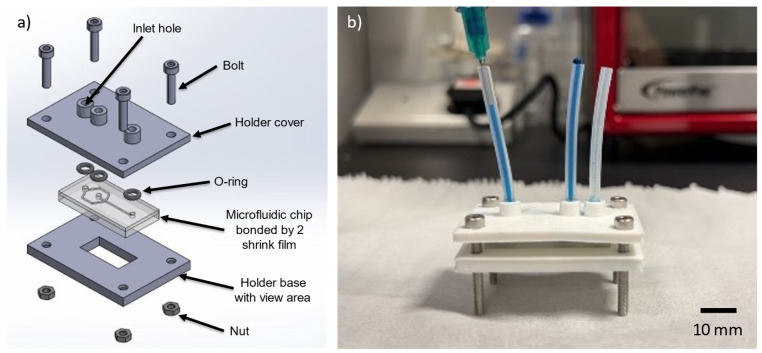
Assembly for the shrink film microfluidic device: (**a**) design of the chip holder; (**b**) assembled microfluidic device with the chip holder.

A chip holder was designed and fabricated to mount the shrink film microfluidic device for experimental use. The chip holder was designed using computer-aided design (CAD) software and fabricated in polylactic acid (PLA) using a 3D printer. The holder was customized to fit the dimensions of the shrink film microfluidic device. The shrinkage ratio of the film was used to determine the size of the holder required to enclose the microfluidic device. The design of the holder included ports for fluid inlets and outlets. The holder provided a secure and stable platform for the microfluidic device, allowing for accurate measurements and observations during experimentation. Figure 7 illustrates both the schematic design of the chip holder assembly, where both the cover and base of the holder were created using 3D printed, and the assembled microfluidic device with the chip holder. The position and numbers of inlets and outlets port can be customized for different shrink film microfluidic chip depending on the microfluidic channel design.

### 3.8. Shrink Film as Mold for PDMS Microfluidic Chip Fabrication

Alternatively, instead of using the shrink film itself as a microfluidic chip, we also demonstrate the use case of using the shrink film as the mold for PDMS fabrication of microfluidic devices. The process involves heating the shrink film to a specific temperature, which causes it to shrink and form a negative impression of the desired microchannel pattern. Figure 6a shows the pre-shrunk film cut-out as a Y-shaped microchannel, while ***b*** shows the shrunk microfluidic channel structure as a negative impression. This negative impression can then be used as a mold for the casting of materials such as PDMS. The resulting microfluidic device can be seen in Figure 6c. The use of shrink film as a mold for casting PDMS eliminated the need for photolithography, resulting in a simplified, rapid, and cost-effective fabrication process. Using shrink film as a mold for PDMS microchannels offers advantages such as cost effectiveness, ease of use, and the ability to produce microchannels with complex geometries.

**Figure 8:**
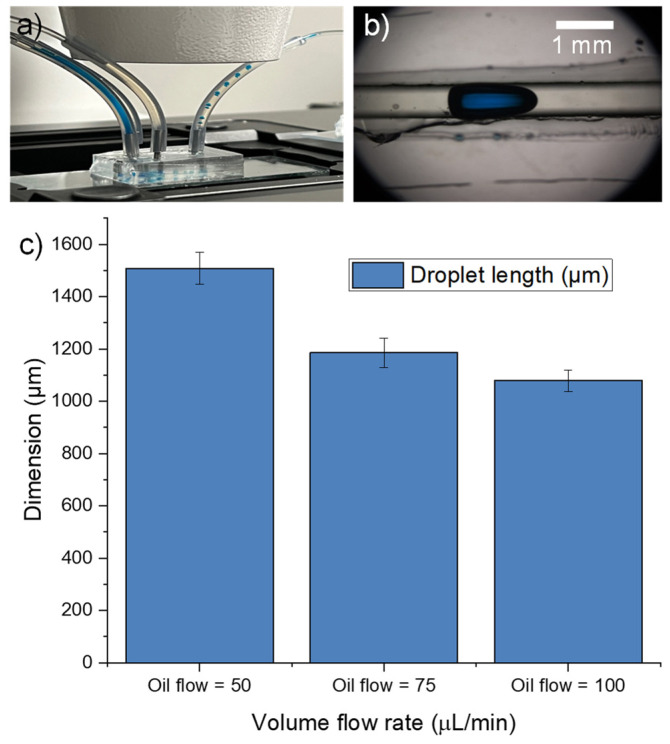
(**a**) Droplet generation by introducing two immiscible fluids (with deionized water with blue dye in the middle inlet, and olive in the side inlets) into the inlet and observing the formation of droplets as they passed through the PDMS microchannel and exit from the outlet. (**b**) Optical micrograph of the droplet generated. (**c**) Plot of droplet length with oil flow rates of 50, 75, and 100 µL/min, respectively, with deionized water fixed at 5 µL/min.

We also conducted an experiment to assess the fabricated PDMS microfluidic chip’s capability for droplet generation by introducing two immiscible fluids (olive oil and deionized water with blue dye) into the inlet and observing the formation of droplets as they passed through the narrow channel section and exit from the outlet (Figure 8a). The results showed that the microfluidic device was able to generate uniform droplets (Figure 8b) with dimensions ranging from 1000 to 1500 μm depending on the flow rates of the fluids. These findings demonstrate the suitability of the fabricated microfluidic device for droplet-based applications in various fields, such as biochemistry, pharmaceuticals, and materials science. Different droplet lengths were observed at varying flow rates. By setting the flow rate of the oil (continuous phase) at 50, 75, and 100 μL/min and the deionized water with blue dye solution (dispersed phase) at 5 μL/min, three distinct droplet lengths were observed. From Figure 8c, the graph shows that there is an inverse proportionality between the oil flow rate and length of the droplets generated. When the continuous phase flow rate (oil) is increased while keeping the dispersed phase flow rate (deionized water with blue dye) constant, the volume of the aqueous phase droplet decreases due to increased shearing force [29], resulting in the observation of smaller droplets. Droplet creation within a microfluidic T-junction is primarily influenced by the interfacial force occurring between the two immiscible liquids. The positioning of the three-phase contact line along the channel’s surface is impacted by factors such as surface energy, channel wall wettability (hydrophobic with contact angle > 90° or hydrophilic with contact angle < 90°), and additional hydrodynamic forces [30].

## 4. Conclusions

In conclusion, this work has demonstrated the feasibility and versatility of using shrink film as a cost-effective and efficient material for the rapid fabrication of microfluidic lab-on-chip devices. Parametric investigations were conducted to explore and optimize the influence of time and temperature on both the shrinkage ratio and the quality of the microfluidic channels. The findings revealed a significant impact of both time and temperature on the shrinkage ratio of the microfluidic channels. In other words, there existed a trade-off between the shrinkage ratio and the quality of the microfluidic channels. Elevated temperatures and longer durations resulted in decreased channel quality due to deformations and irregularities. From the parametric studies involving various heating time (1–10 min) and temperatures (90–250 °C), we determined that the ideal heating conditions of 150 °C with a baking time of 4 min would result in the optimal size for microfluidic device fabrication. Under these ideal conditions, the shrink film contracts ~2.5 times in the X- and Y-axes, while the Z-axis thickens by a factor of ~5.8 times. Moreover, this investigation characterized the attributes of the shrink film, such as the wettability, and the smallest achievable feature size and cross-sectional geometries through the CAMEO 4 automated cutting machine and manual engraving. This study successfully attained a minimum feature size of 200 microns. However, this was constrained by the collapsing of channel sidewalls during the shrinking process, causing blockages in the microchannel. In addition, two approaches were examined to assess the applicability of shrink film in microfluidic device manufacturing: employing the shrink film itself as the microchannel and thermally bonding it with another piece of unpatterned shrink film, and utilizing the shrink polystyrene film as a mold or negative impression for PDMS casting. Both methods were demonstrated to be viable. These findings underscore the potential of employing shrink film in the fabrication of microfluidic channels, presenting a promising alternative to conventional photolithography methods.

## Figures and Tables

**Table 1:** Advantages and disadvantages of shrink film microfluidics [18].

Advantages	Disadvantages
Cost effective	Thermal sensitivity
Easy to fabricate	Limited mechanical strength
Optical transparency	Limited scalability

## Data Availability

The data presented in this study are openly available online at https://doi.org/10.21979/N9/JM9OLV (accessed on 31 January 2024).
